# Data on the estimating the risk of cancer due to some common radiographs in Tehran city

**DOI:** 10.1016/j.dib.2018.08.208

**Published:** 2018-09-06

**Authors:** Mohammad Mirdoraghi, Amin Banaei, Jafar Fatahi Asl

**Affiliations:** a Department of Radiology and Radiotherapy, School of Allied Medicine, Tehran University of Medical Sciences, Tehran, Iran; b Department of Radiology, Faculty of Paramedicine, Aja University of Medical Sciences, Tehran, Iran; c School of Paramedicine, Department of Radiologic Technology, Ahvaz Jundishapur University of Medical Sciences, Ahvaz, Iran

**Keywords:** Common radiographs, Risk estimation, Radiology, PCXMC 2.0, Cancer

## Abstract

The purpose of the data is to estimate the excessive risk of cancer due to some common radiographs in Tehran. The data were collected in 8 radiology centers in Tehran city and on 283 patients with eight radiographic views. To obtain the data, PCXMC 2.0 based on Monte Carlo calculations, has been used to calculate the effective dose of each organ, and annual effective dose. The effective dose, cumulative effective dose, number of radiographs per year and excessive cancer risk due to the type of radiographs calculated. The additional risk of lethal cancer resulting from these radiographs in the target population is about 14.81 cases of the total population of Tehran city in one year.

**Specifications table**

**Table:** 

Subject area	Radiation protection and radiation biology.
More specific subject area	Calculating the risk of cancer due to some common radiographs in Tehran city.
Type of data	Tables
How data was acquired	The data on the cumulative effective dose of the population was calculated by PCXMC 2.0 software, and then the excessive risk of cancer was determined by standard equations.
Data format	Raw, Analyzed.
Experimental factors	The cumulative effective dose of the population was analyzed according to the equations for the excessive risk of radiation-induced cancer.
Experimental features	The excessive risk of radiation-induced cancer was determined.
Data source location	Radiology wards in educational hospitals affiliated to AJA university, Tehran province, Iran.
Data accessibility	The data are available with this article.

**Value of the data**

• The data show that the number of excessive cancers due to the common radiographs for the entire population of Tehran city is 14.81 cases per year. Therefore, data can be used to reduce the number of unnecessary radiographs and ultimately reduce the amount of cancer in Tehran city.
• The data is useful in demonstrating that the optimization of exposure factors including FOV, KVP, MAS and FSD could diminish the effective dose of patients in common radiographs. Therefore, more researches is needed to adjust the mentioned parameters to decrease the excessive risk of radiation-induced cancer in the current population.
• The data can be used to show that a specialist in radiation protection and radiation biology should supervise the radiology ward in order to monitor patient safety, quality control and quality assurance of radiology machines; consequently, the effective dose and the number of excessive cancers due to the common radiographs would be decreased.

## 1 Data

In Table 1, the ESD, the mean of the exposure parameters used for each type of radiography is presented.

**Table 1:** Exposure parameters in the common radiology procedures and ESD.

**Radiology procedure**	**Mean KVP**	**Mean MAS**	**FSD (cm)**	**ESD (mGy)**
chest (Anterior-Posterior view)	65	20	180	0.15
pelvic (Anterior-Posterior view)	85	48	100	5.4
abdomen (Anterior-Posterior view)	64	44	100	2.7
skull (Anterior-Posterior view)	73	17	100	2.2
Thoracic spine (Anterior-Posterior view)	76	40	100	4.6
lumbar (Anterior-Posterior view)	76	55	100	5.8
lumbar spine (Lateral view)	85	69	100	6.2
Thoracic spine (Lateral view)	80	54	100	5.1

Table 2 shows the effective dose, cumulative effective dose, the number of radiographs per year, and excessive risk of cancer.

**Table 2:** Effective dose, cumulative effective dose, number of radiographs per year, and excessive risk of cancer from each radiography in the population of Tehran city in a year.

**Radiology Procedure**	**the annual number of X-ray examinations per 1000 population**	**Number of radiographs in the total population**	**The average effective dose**	**Accumulative effective dose (Man-Sievert)**	**The number of excessive cancers for the entire population of Tehran in one year**
Chest	90.12	784,172	0.028	21.95	1.09
abdomen (Anterior-Posterior view)	11.28	98,065	0.87	85.3	4.26
pelvic (Anterior-Posterior view)	14.91	129,623	0.61	79.07	3.95
lumbar spine	24.9	216,473	0.385	83.34	4.167
skull	25.77	224,036	0.05	11.20	0.56
Thoracic spine	7.61	66,159	0.24	15.87	0.79

## 2 Experimental design, materials and methods

### 2.1 Data collection

Data were collected on the 283 patients (65% male, 35% female) in eight common radiographs including, chest (Anterior-Posterior view), pelvic (Anterior-Posterior view), abdomen (Anterior-Posterior view), skull (Anterior-Posterior view), thoracic spine (Anterior-Posterior view), Thoracic spine (Lateral view), lumbar spine (Anterior-Posterior view), lumbar spine (Lateral view). At first, the accuracy tests of measuring half layer absorption, the accuracy of voltage of X-ray tube, time, Milliampere-seconds (mAs) were performed using Barracuda dosimeter (Multi-Purpose Detector). Then, the output of the X-ray machine was recorded at a distance of 100 cm and a voltage of 80 kV for 3 times in order to decrease the potential error. Also, all radiation parameters (voltage of tube, milliampere-seconds, focus to skin distance (FSD), filter thickness, field-of-view size (FOV) were recorded for all patients. To estimate Entrance Surface Dose (ESD) of the patients, the following equation used [1], [2].

$$ESD=A\times {\left(KV/80\right)}^{2}\times mAs\times B/FSD^{2}$$

A=The output of tube at a KV of 80 at 1 m distance from X-ray tube.
B=Correction factor based on IAEA report no. 457.
MAs=Milliampere-seconds applied.
FSD=focus to skin distance (cm).

### 2.2 Calculating the effective dose

In order to estimate the annual effective dose and effective doses of organs the radiation parameters i.e. KV, MA-S, FSD, filter thickness, FOV entered in the PCXMC 2.0 software, manufactured by STUK–Radiation and Nuclear Authority, Helsinki, Finland.

### 2.3 Risk assessment

In order to estimate the excessive risk of radiation-induced cancer, the cumulative dose of the study population is required. The number of radiographs per year in the current society was extracted from previous studies [3], [4], [5]. Data on the cumulative effective dose for eight radiographs using of scientific relationships based on the acceptance of linear no-threshold model were obtained. Then, the risk of excessive cancer for each radiography was determined by standard equations [6], [7].

The population of Tehran city is around 8,693,706 by the 2016 census [8].

Assuming that the frequency of radiographs in Tehran city is equal to the radiographic frequency in the country [9], the annual cumulative effective dose can be calculated for a specific radiography. Consequently, according to the following formula, the excessive risk of lethal cancer is obtained per 100,000 people [10], [11].

Excessiveriskoflethalcancer=%5×(Man−Sievert)

## Conflict of interest

None to declare.
