# Supplementary material for: Data on the estimating the risk of cancer due to some common radiographs in Tehran city
Source: Data Brief. 2018 Sep 6;20:1269–73. doi: 10.1016/j.dib.2018.08.208 (PMC6143744; doi:10.1016/j.dib.2018.08.208)
Supplement: Supplementary file 1 — Supplementary material. [file mmc1.docx]

To the editor, 24^th^ August 2018

**Data in Brief**

Dear editor,

There is no any conflict of interest for the manuscript entitled, “**Data on the Estimating the Risk of Cancer Due to Some Common Radiographs in Tehran City.*”*** All authors declare that.

Thank you in advance for receiving our manuscript to review and your valuable time. Looking forward to hear from you soon.

Sincerely Yours,
*Mohammad Mirdoraghi*
